# Overexpression of *Populus trichocarpa CYP85A3* promotes growth and biomass production in transgenic trees

**DOI:** 10.1111/pbi.12717

**Published:** 2017-06-17

**Authors:** Yan‐Li Jin, Ren‐Jie Tang, Hai‐Hai Wang, Chun‐Mei Jiang, Yan Bao, Yang Yang, Mei‐Xia Liang, Zhen‐Cang Sun, Fan‐Jing Kong, Bei Li, Hong‐Xia Zhang

**Affiliations:** ^1^ College of Agriculture Ludong University Yantai China; ^2^ National Key Laboratory of Plant Molecular Genetics Shanghai Institute of Plant Physiology and Ecology Chinese Academy of Sciences Shanghai China; ^3^ University of Chinese Academy of sciences Beijing China; ^4^ MLR Key Laboratory of Saline Lake Resources and Environments Institute of Mineral Resources CAGS Beijing China

**Keywords:** biomass production, brassinosteroids, *CYP85A3*, poplar, transgenic plant, xylem differentiation

## Abstract

Brassinosteroids (BRs) are essential hormones that play crucial roles in plant growth, reproduction and response to abiotic and biotic stress. In *Arabidopsis*, AtCYP85A2 works as a bifunctional cytochrome P450 monooxygenase to catalyse the conversion of castasterone to brassinolide, a final rate‐limiting step in the BR‐biosynthetic pathway. Here, we report the functional characterizations of *PtCYP85A3*, one of the three *AtCYP85A2* homologous genes from *Populus trichocarpa*. *PtCYP85A3* shares the highest similarity with *AtCYP85A2* and can rescue the retarded‐growth phenotype of the *Arabidopsis cyp85a2‐2* and tomato *d* ^*x*^ mutants. Constitutive expression of *PtCYP85A3*, driven by the cauliflower mosaic virus 35S promoter, increased the endogenous BR levels and significantly promoted the growth and biomass production in both transgenic tomato and poplar. Compared to the wild type, plant height, shoot fresh weight and fruit yield increased 50%, 56% and 43%, respectively, in transgenic tomato plants. Similarly, plant height and stem diameter increased 15% and 25%, respectively, in transgenic poplar plants. Further study revealed that overexpression of *PtCYP85A3* enhanced xylem formation without affecting the composition of cellulose and lignin, as well as the cell wall thickness in transgenic poplar. Our finding suggests that *PtCYP85A3* could be used as a potential candidate gene for engineering fast‐growing trees with improved wood production.

## Introduction

Brassinosteroids (BRs) are the sixth class of plant‐specific steroidal hormones that are involved in many bioprocesses in plants, including cell division and elongation, xylem differentiation, seed germination, vegetative growth, apical dominance and photomorphogenesis (Azpiroz *et al*., 1998; Choe *et al*., 1998; Clouse *et al*., 1996; Fujioka *et al*., 1997; Yamamoto *et al*., 2007). Like other plant hormones, BRs also protect plants from a variety of environmental stresses, such as high and low temperature, drought, salinity, herbicidal injury and pathogen attack (Khripach *et al*., 2000; Krishna, 2003).

The biosynthetic pathways of BRs have been established by feeding deuterio‐labelled compounds of possible BR intermediates to cultured cells of *Catharanthus roseus* and by the careful analyses of their bioconversion products by gas chromatography–mass spectrometry (GC‐MS; Sakurai, 1999). These experiments revealed that brassinolide (BL) is biosynthesized from campesterol (CR) via two parallel branched pathways, namely the early and late C6‐oxidation pathways (Choi *et al*., 1997). However, the predominance of either pathway varies among different plant species and tissues. For example, both pathways occur in pea (Nomura *et al*., 1999) and *Arabidopsis* (Fujioka *et al*., 1997), whereas the late C6‐oxidation pathway is predominant in tomato (Bishop *et al*., 1999).

In *Arabidopsis*, five crucial BR‐specific biosynthesis genes have been identified: DEETIOLATED2 (DET2; Li *et al*., 1996), CONSTITUTIVE PHOTOMORPHOGENESIS AND DWARFISM (CPD; Szekeres *et al*., 1996), DWARF4 (DWF4; Choe *et al*., 1998), BR‐6‐oxidase1 (BR6ox1; Bishop *et al*., 1999) and ROTUNDFOLIA3 (ROT3; Kim *et al*., 1998). Several BR metabolism enzymes, including sulphotransferases ST1 and ST4a (BNST3/4 in *Brassica napus*; Marsolais *et al*., 2004, 2007; Rouleau *et al*., 1999), a UDP glucosyltransferase UGT73C5 (Poppenberger *et al*., 2005), P450 hydroxylases PHYB‐4 ACTIVATION TAGGED SUPRESSOR 1 (BAS1) and SUPRESSOR OF PHYB‐4 7 (SOB7; Turk *et al*., 2005), a BAHD acyltransferase‐like protein BRASSINOSTEROID INACTIVATOR 1 (BIA1; Roh *et al*., 2012), a protein having acyl‐CoA ligase activity (PIZZA; Schneider *et al*., 2012), and a gene encoding a dihydroflavonol 4‐reductase (DFR) and anthocyanidin reductase (BAN)‐like protein (BEN1; Yuan *et al*., 2007), have been characterized. Mutants with reduced activity of the above BR‐biosynthetic enzymes or increased BR metabolic enzymes generally exhibit altered phenotypes such as severe dwarfism, round and dark‐green leaves, delayed senescence, reduced male fertility, and defective skotomorphogenesis in darkness (Choe *et al*., 1998; Chory *et al*., 1991; Clouse *et al*., 1996; Kauschmann *et al*., 1996; Li and Chory, 1997; Li *et al*., 1996; Noguchi *et al*., 1999; Szekeres *et al*., 1996).

Since the discovery of brassinolide (BL) from the pollens of *Brassica napus* (Grove *et al*., 1979), more than 70 BR compounds have been isolated from various plant species (Bajguz, 2007). The most active types of brassinosteroids are brassinolide (BL) and castasterone (CS), which are catalysed by members of the CYP85A family of cytochrome P450 monooxygenases. To date, one CYP85A in *Oryza sativa* (CYP85A1; Hong *et al*., 2002; Mori *et al*., 2002), *Vitis vinifera* (BR6ox1; Symons *et al*., 2006), *Hordeum vulgare* (DWARF; Gruszka *et al*., 2011), *Zea mays* (BRD1, BRASSINOSTEROID DEFICIENT DWARF 1; Makarevitch *et al*., 2012) and *Brachypodium distachyon* (BRD1; Xu *et al*., 2015), and two CYP85As in *Arabidopsis thaliana* (CYP85A1 and CYP85A2; Kim *et al*., 2005; Shimada *et al*., 2001, 2003), *Solanum lycopersicum* (CYP85A1 and CYP85A3; Bishop *et al*., 1996, 1999; Nomura *et al*., 2005) and *Pisum sativum* (CYP85A1 and CYP85A6; Jager *et al*., 2007) have been isolated. In the regulation of biologically active brassinosteroid (BR) levels in plant, C‐6 oxidation genes play a key role. Overexpression of *AtCYP85A2* significantly enhanced the vegetative and reproductive growth of *Arabidopsis*. Compared to the wild type, transgenic plants possessed larger rosette leaves with longer petioles, taller and multibranched stems bearing larger siliques with longer pedicels, resulting in a 30% increase in the seed‐per‐silique number (Kim *et al*., 2005). Overexpression of *AtDWF4* in *Arabidopsis* (*AOD4*) and tobacco (*TOD4*) also improved the biomass of transgenic plants (Choe *et al*., 2001). Compared to the controls, an over 35% increase in *AOD4* lines and over 14% increase in *TOD4* lines were observed in the inflorescence heights of transgenic plants. A 59% increase in seed number was also achieved due to the more than doubled branch and silique production in *AOD4* transgenic plants. Similar results were also observed in transgenic rice expressing *DWF4s* from maize (*Zm‐CYP*), rice (*Os‐CYP*) and *Arabidopsis* (*At‐gCYP*). The seeds of transgenic rice expressing *Zm‐CYP*,* Os‐CYP* or *At‐gCYP* were larger and heavier than those of the wild type. The up to 19% increase in shoot height and 28% increase in tiller number led to an up to 42% biomass increase in *Zm‐CYP* and *At‐gCYP* transgenic plants (Wu *et al*., 2008).

Although the functions of CYP85A family proteins have been well studied in herbaceous plants, their possible roles in wooden tree plants, especially in xylem development, still remain unknown. In this work, we isolated *PtCYP85A3* from *Populus* and investigated its role in shoot growth and wood development. We found that *PtCYP85A3* worked as a functional homologue of *AtCYP85A2* and *SlCYP85A1*. Overexpression of *PtCYP85A3* in aspen enhanced the biosynthesis of BRs, leading to promoted growth rate and wood production in transgenic plants. Further studies revealed that transcriptions of some xylem‐related MYB transcription factors and cellulose synthase genes were increased.

## Results

### PtCYP85A3 encodes a putative BR C‐6 oxidase in *Populus*

**Figure 1:**
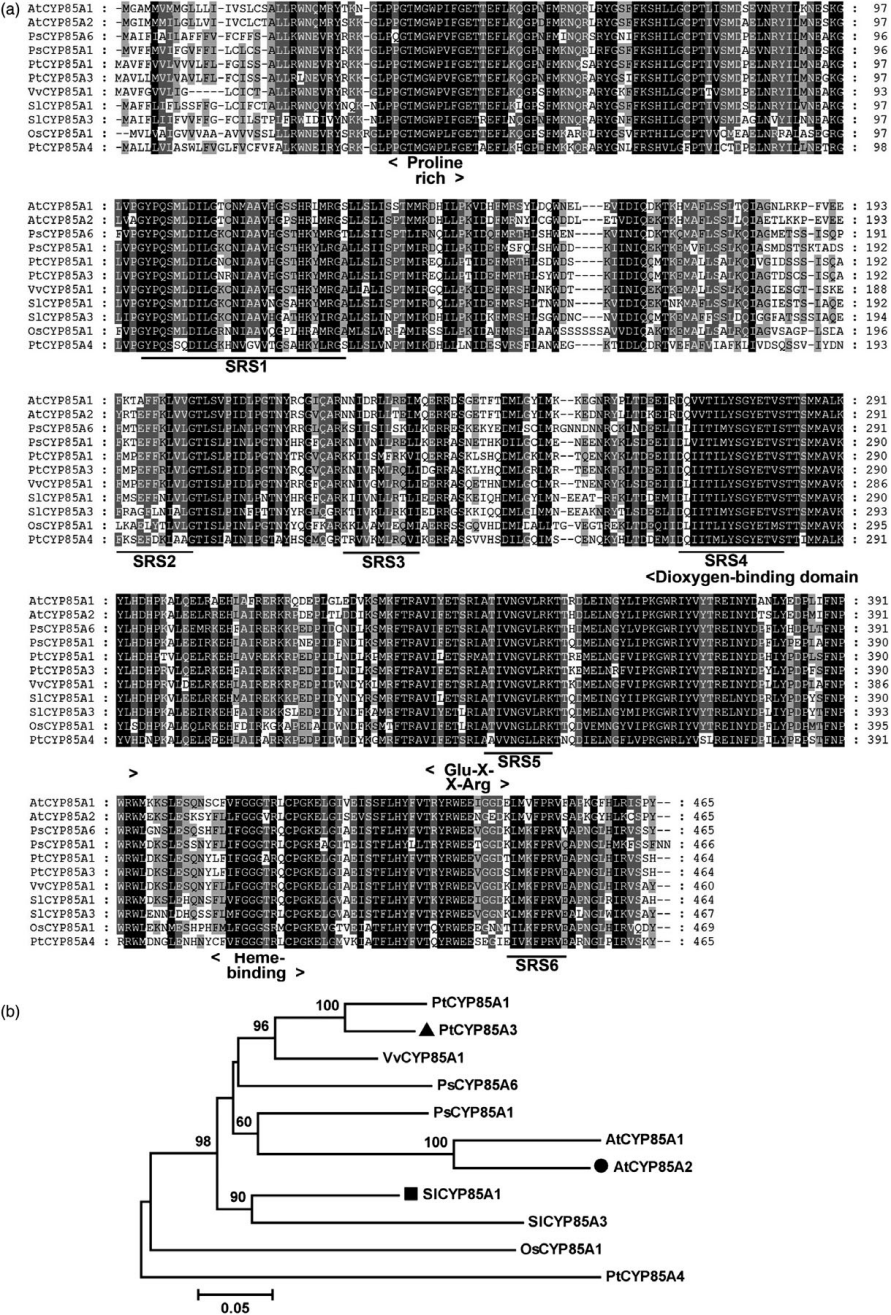
Amino acid sequence alignment and phylogenetic analysis of CYP85A clan P450 members in different plants. (a) Comparison of cytochrome P450 monooxygenase proteins from *Arabidopsis thaliana* AtCYP85A1 (BAB60858) and AtCYP85A2 (BAC55065), *Pisum sativum* PsCYP85A1 (BAF56235) and PsCYP85A6 (BAF56236), *Solanum lycopersicum* SlCYP85A1 (DWARF, AAB17070) and SlCYP85A3 (BAD98244), *Oryza sativa* OsCYP85A1 (BAC45000), *Vitis vinifera* VvCYP85A1 (ABB60086), *Populus trichocarpa* PtCYP85A1 (EEE85904), PtCYP85A3 (EEF10243) and PtCYP85A4 (EEF02250). Residues are highlighted in black, dark grey and light grey according to the level of conservation. (b) Phylogenetic tree of CYP85A clan P450 members in different plants. Phylogram in which the branch lengths are proportional to sequence divergence was conducted with MEGA 5.

To understand the possible functions of CYP85A family proteins in wooden plants, we performed a BLAST search in the Joint Genome Initiative poplar database (JGI, *Populus trichocarpa* genome portal v1.1; http://genome.jgi-psf.org/Poptr1_1/Poptr1_1.home.html) and identified three homologues in the *Populus* genome base on the CDS of *SlCYP85A1*, named as *PtCYP85A1* (EEE85904), *PtCYP85A3* (EEF10243) and *PtCYP85A4* (EEF02250). Among them, PtCYP85A3 shares the highest sequence identity with AtCYP85A2 (91.40%) and SlCYP85A1 (94.18%; Figure 1a, b). Similar to AtCYP85A2 and SlCYP85A1, PtCYP85A3 is characterized by a Pro‐rich and dioxygen‐binding domain, a Glu‐X‐X‐Arg motif and a Heme‐binding domain, which are highly conserved in most CYP450 proteins.

### Expression pattern of *PtCYP85A3* in *Populus*

**Figure 2:**
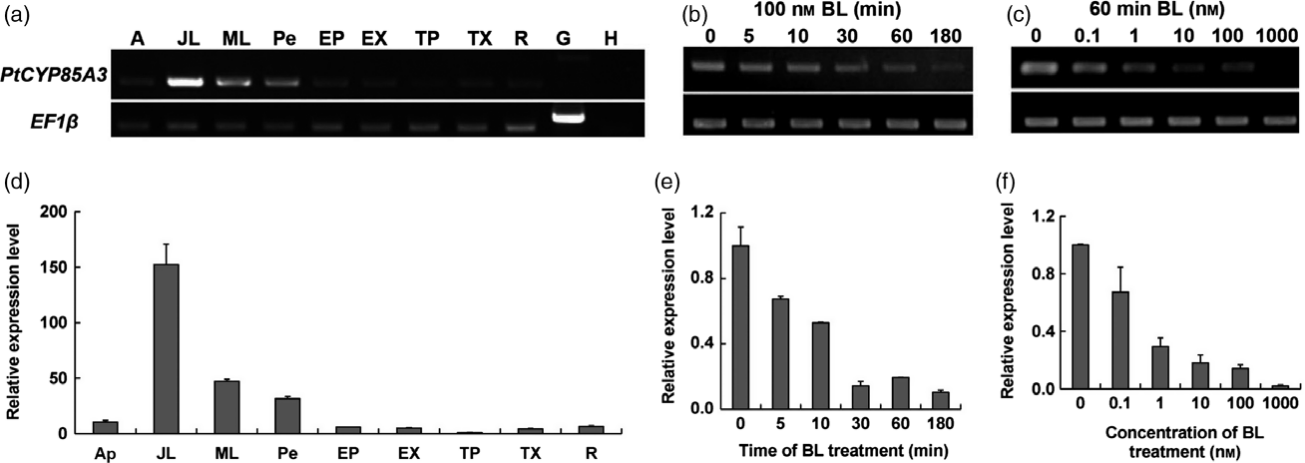
Expression pattern of *PtCYP85A3* in poplar. (a, d) RT‐PCR and qRT‐PCR analysis of *PtCYP85A3* gene in different tissues of 3‐month‐old Shanxin yang. cDNA derived from indicated tissues was used as template for PCR with gene‐specific primes. Products from indicated PCR cycles were visualized by agarose gel electrophoresis and ethidium bromide staining. The elongation factor gene *EF1*β was analysed as an internal control. A, apical buds; JL, juvenile leaves; ML, mature leaves; Pe, petiole; EP, phloem of elongating stem; EX, xylem of elongating stem; TP, phloem of thickening stem; TX, xylem of thickening stem; R, root; G, genome DNA; H, H_2_O. (b, e) RT‐PCR and qRT‐PCR analysis of *PtCYP85A3* transcript in response to 100 nm BL treatment for 0, 5, 10, 30, 60, 180 min. (c, f) RT‐PCR and qRT‐PCR analysis of *PtCYP85A3* transcript in the presence of 0, 0.1, 1, 10, 100 and 1000 nm BL for 30 min.

To further elucidate the roles of *PtCYP85A3*, we isolated total RNA from different tissues of 3‐month‐old Shanxin yang and analysed the transcript abundance of *PtCYP85A3* by semiquantitative RT‐PCR and quantitative real‐time RT‐PCR with *PtCYP85A3*‐specific primers (Table S1). We observed that *PtCYP85A3* was ubiquitously expressed in poplar plants and preferentially expressed in juvenile leaf (Figure 2a, d). In *Arabidopsis*, the transcription of many BR‐biosynthetic genes was suppressed when plants were treated by BR spraying. We found that expression of *PtCYP85A3* was also rapidly depressed by BL in a dosage‐dependent manner in Shanxin yang (Figure 2b, c, e, f). Therefore, the biosynthesis of BR can be negatively feedback‐regulated by BR signal so that the equilibrium of endogenous BRs can be achieved for the finely regulated growth and development of plants.

### Subcellular localization of PtCYP85A3

**Figure 3:**
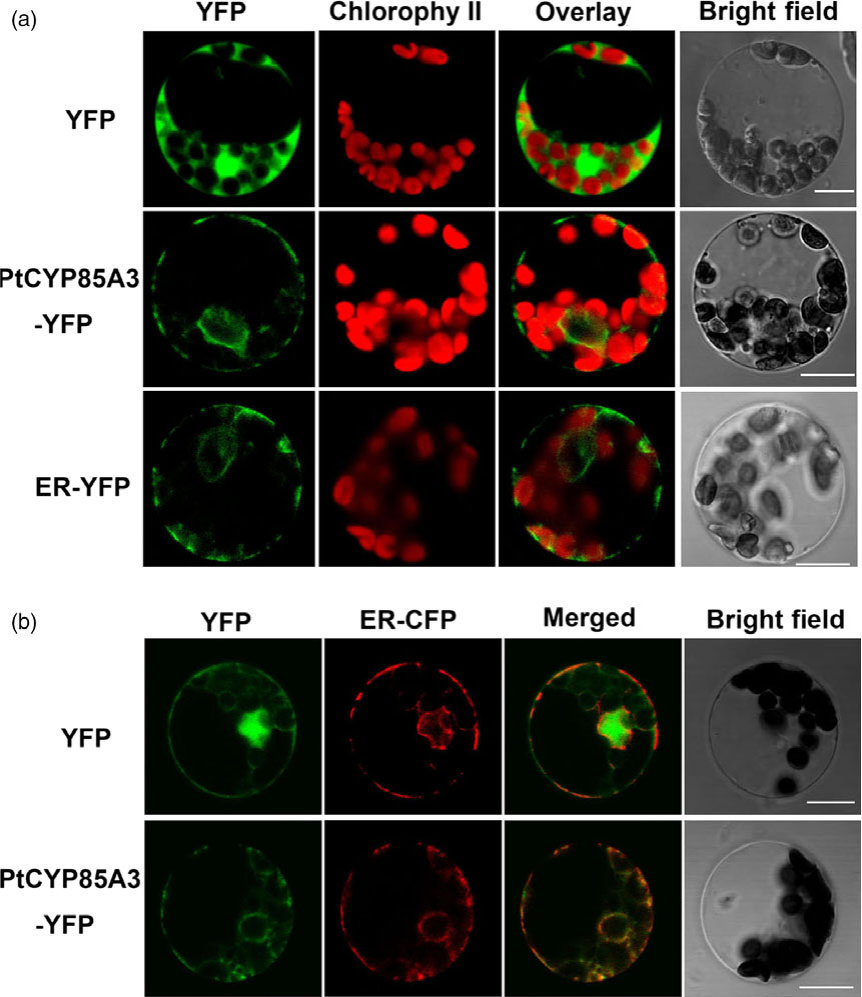
PtCYP85A3 is targeted to endoplasmic reticulum. (a) Confocal microscopic analysis of YFP signals from poplar mesophyll protoplasts transiently expressing a free YFP, PtCYP85A3‐YFP or ER‐YFP as indicated. Scale bars = 5 μm. (b) An ER‐specific marker ER‐CFP was co‐transformed with YFP alone or with PtCYP85A3‐YFP construct into poplar mesophyll protoplasts and then analysed by confocal microscopy. Free YFP signal (green) is largely separate from ER‐CFP signal (red). PtCYP85A3‐YFP co‐localized with the ER‐CFP marker as indicated by the yellow colour in the merged image. Scale bars = 5 μm.

Previous studies showed that eukaryotic cytochrome P450 enzymes were generally located in the endoplasmic reticulum (ER; Schuler, 1996). In addition, BR‐biosynthetic enzyme DWF4 is also located in the endoplasmic reticulum (Kim *et al*., 2006). Thus, it is possible that the rate‐determining enzyme in BR biosynthesis is targeted to a similar location. To determine the subcellular localization of PtCYP85A3, a PtCYP85A3‐YFP (yellow fluorescent protein) fusion protein, together with the control plasmid pA7‐YFP and the ER marker ER‐YFP (Nelson *et al*., 2007), was transiently transfected into poplar mesophyll cell protoplasts and visualized under a confocal laser scanning microscopy. Co‐transformation of ER‐CFP (cyan fluorescent protein) in a combination with PtCYP85A3‐YFP was also performed. As we have expected, the fluorescence of PtCYP85A3‐YFP perfectly overlapped with that of ER‐CFP, revealing that PtCYP85A3‐YFP was indeed targeted to the ER organelle (Figure 3a, b). As *PtCYP85A3* catalysed the final and rate‐limiting step in the BR‐biosynthetic pathway, the subcellular localization of PtCYP85A3 in endoplasmic reticulum is consistent with the hypothesis that BRs were synthesized in the endoplasmic reticulum of plant cells.

### Constitutive expression of *PtCYP85A3* promotes shoot elongation, plant size and overall yield in tomato

To understand the exact function of PtCYP85A3 in plants, we isolated the coding sequence of *PtCYP85A3* and constructed a plant expression vector pCAMBIA2301 or pCAMBIA1301 under the control of the cauliflower mosaic virus 35S (CaMV 35S) promoter (Figures S1a and S2a). The expression of *PtCYP85A3* driven by the CaMV 35S promoter in *Arabidopsis* (*cyp85a2‐2*) and tomato (*d* ^*x*^) mutants restored them to the wild type (Col‐0) and LA2838 phenotype, confirming that PtCYP85A3 is a functional homologue of AtCYP85A2 and SlCYP85A1 (Figure S1b‐c). Then, *PtCYP85A3* was introduced into the Micro‐Tom tomato, using an *Agrobacterium*‐mediated transformation method as described previously (Zhang and Blumwald, 2001). Four independent transgenic lines were chosen from PCR‐ and semi‐quantitative RT‐PCR confirmed regenerants and planted in pots to obtain homozygous seeds (Figure S2b, c). All *PtCYP85A3* transgenic plants showed elongated shoots and bigger plant size compared with that of the wild‐type and the vector control plants (Figure S2d). Detailed comparison between WT and transgenic plants showed that plant height, fresh shoot weight and fruit yield increased 50%, 56% and 43%, respectively, in transgenic tomato plants (Table S2).

### Generation and molecular confirmation of transgenic poplar plants overexpressing *PtCYP85A3*

**Figure 4:**
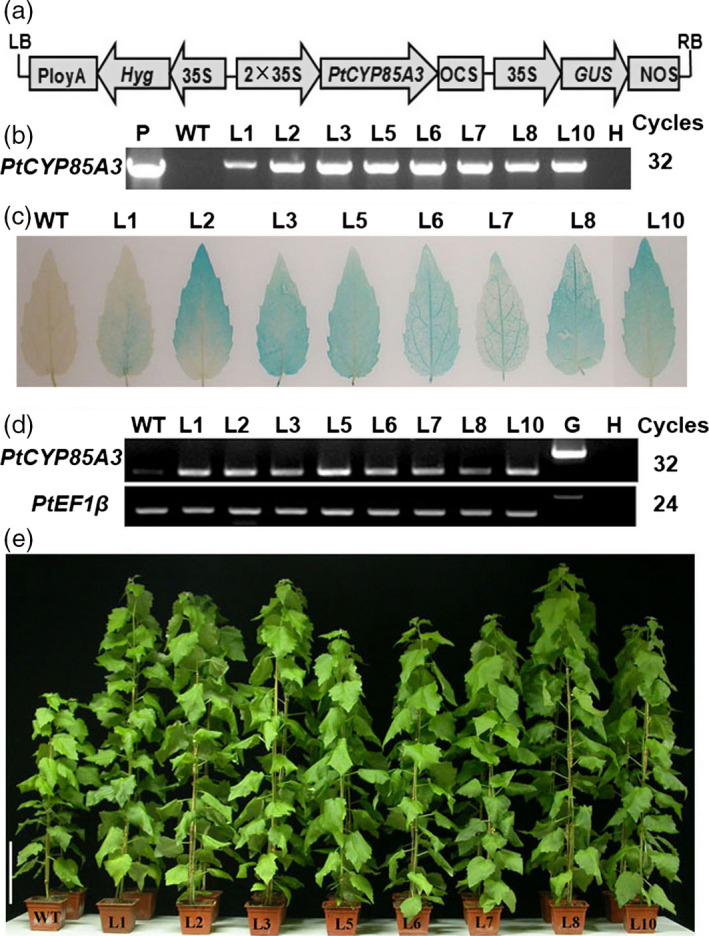
Molecular analyses of *PtCYP85A3* transgenic trees. (a) Schematic map of pCAMBIA1301‐*PtCYP85A3* construct. Expression of *PtCYP85A3* is driven by the cauliflower mosaic virus 35S promoter. (b) PCR analysis of wild type and different independently regenerated transgenic lines. P: plasmid; H: water; WT: wild type; L1‐10: different transgenic lines. (c) GUS staining of *PtCYP85A3* transgenic plants. (d) RT‐PCR analysis of wild type and different transgenic lines. H: water; WT: wild type; L1‐10: different transgenic lines. (e) Phenotypes of wild type and eight independent *PtCYP85A3* transgenic lines grown in glasshouse for 9 weeks. Bar = 15 cm.

Based on the observation that *PtCYP85A3* can functionally complement the *Arabidopsis* (*cyp85a2‐2*) and tomato (*d* ^*x*^) mutants and significantly increase the growth and fruit yield in tomato, we postulated that overexpression of *PtCYP85A3* in *Populus* may promote the growth and wood formation of transgenic plants. To evaluated the potential value of *PtCYP85A3* in improving biomass production in woody plants, we introduced the pCAMBIA1301 construct containing the coding sequence of *PtCYP85A3* into the genome of the aspen hybrid clone Shanxin yang (*Populus davidiana* × *P. bolleana*) by *Agrobacterium*‐mediated transformation (Figure 4a). A total of over 50 independently regenerated hygromycin‐resistant lines were obtained. The integration of *PtCYP85A3* into the poplar genome was confirmed by PCR analyses (Figure 4b). Further analysis by GUS staining (Figure 4c) and semi‐quantitative RT‐PCR (Figure 4d) confirmed the overexpression of *PtCYP85A3* in all the selected transgenic lines. Therefore, three representative lines (L3, L5 and L8) were selected for subsequent analyses (Figure 4e).

### *PtCYP85A3* significantly enhances shoot growth and biomass production

**Figure 5:**
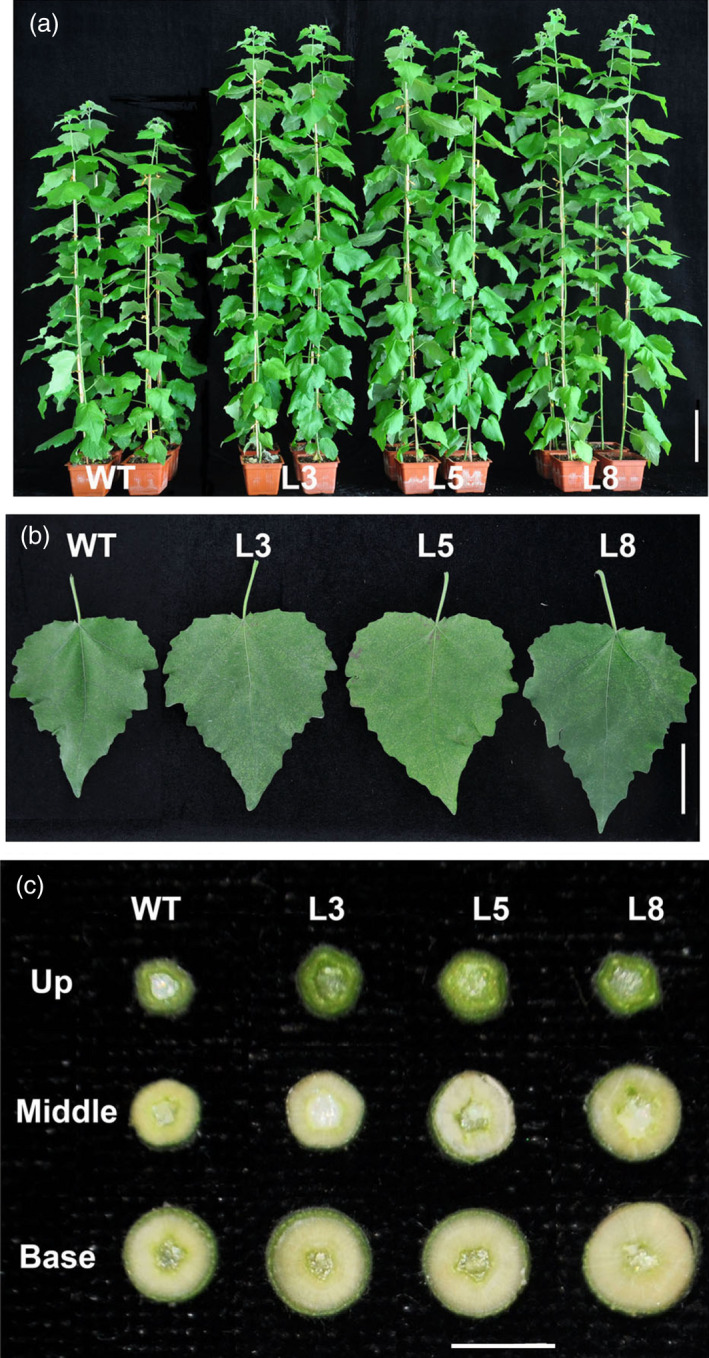
Growth comparisons of wild‐type and *PtCYP85A3* transgenic plants. (a) Phenotype of 3‐month‐old wild‐type and transgenic plants (lines 3, 5 and 8). Bar=10 cm. (b) Leaf phenotypes of 3‐month‐old wild‐type and transgenic plants (lines 3, 5 and 8). Bar=5 cm. (c) The cross sections of the stems from different parts of 3‐month‐old wild‐type and transgenic plants (lines 3, 5 and 8). Bar = 5 mm.

**Figure 6:**
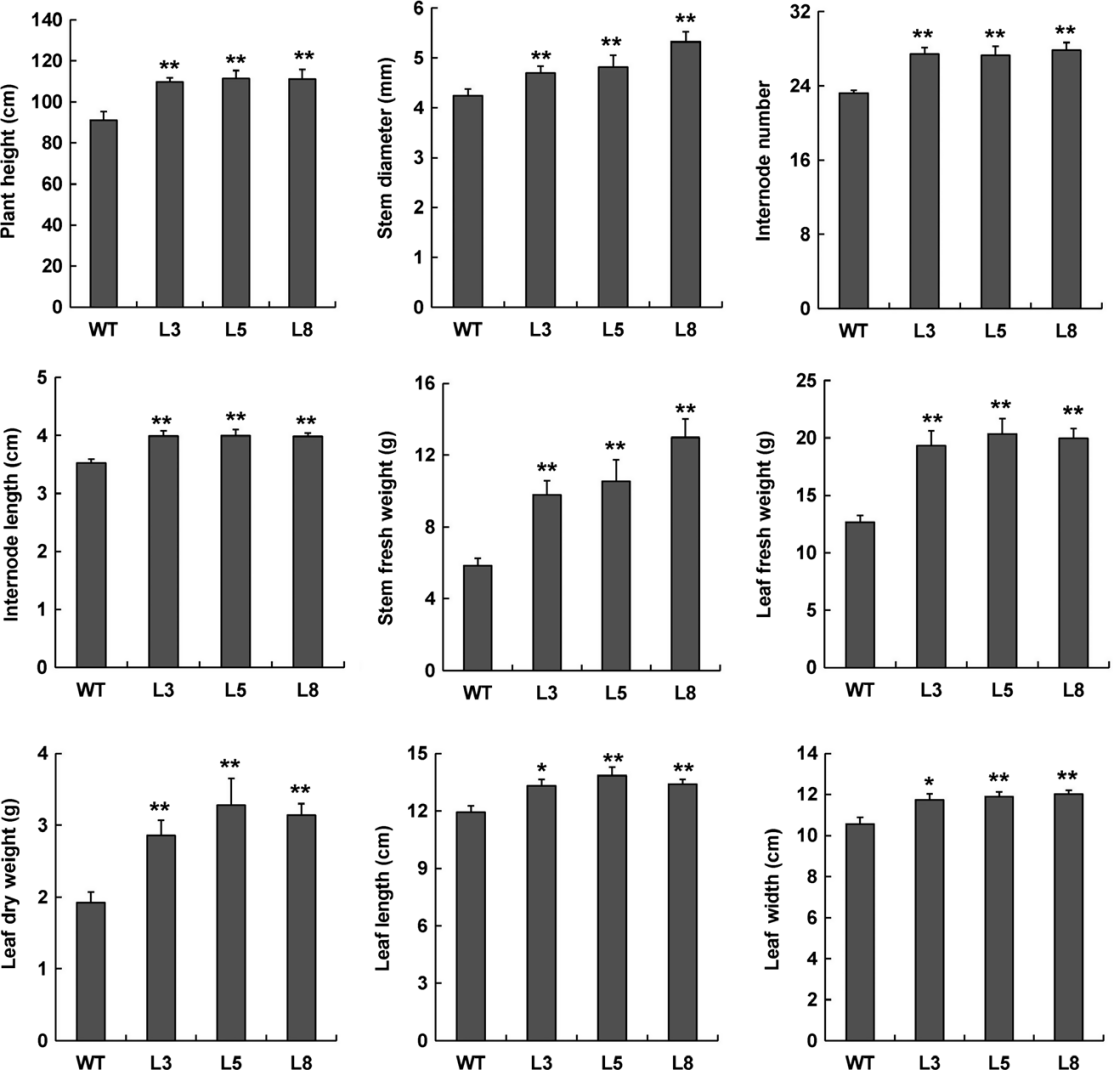
Overexpression of *PtCYP85A3* in poplar promotes plant height, stem diameter and shoot biomass. Five individual plants of WT and each transgenic line were analysed. * and ** indicate significant differences in comparison with WT at *P *< 0.05 and *P *< 0.01, respectively (Student's *t*‐test).

**Figure 7:**
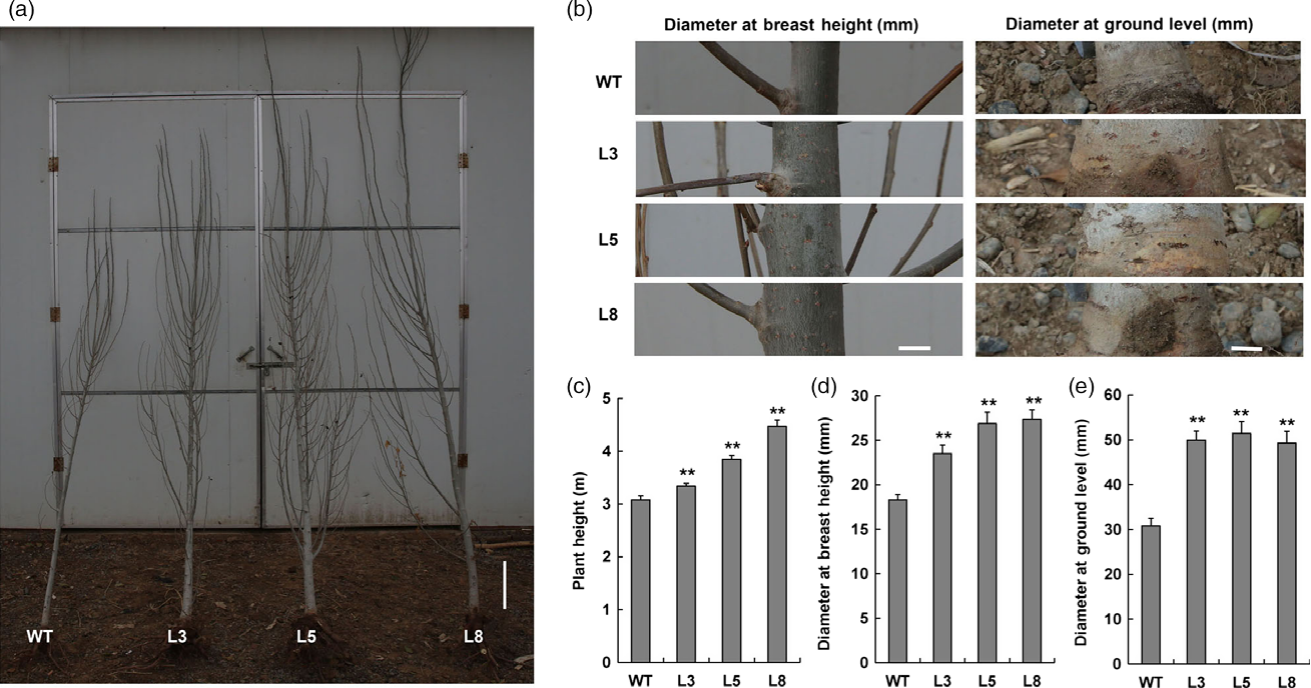
Field trial of transgenic plants. (a, b) Phenotypes of 2‐year‐old wild‐type and transgenic plants (lines 3, 5 and 8) grown in the field. Plant heights and stem diameters at breast and ground levels were shown. Bar=0.3 m. (c–e) Statistical analyses of plant height and stem diameter at breast and ground levels. Bar = 10 mm. ** indicates significant differences in comparison with WT at *P *< 0.01 (Student's *t*‐test).

To determine whether overexpression of *PtCYP85A3* would affect the normal growth and development of transgenic plants, we grew them on MS medium and in glasshouse to examine their phenotypes. As shown in Figure S3, after 1 month on MS medium, transgenic poplar plants showed a fast‐growing phenotype with taller plant height and longer internode length compared to the wild‐type control (Figure S3a, c, d). Same phenotype was also observed on those grown in the glasshouse (Figure S3b). After grown in the glasshouse for 3 months, more significant growth phenotype differences were observed between WT and transgenic plants. Transgenic plants (lines L3 L5 and L8) grew more rapidly than did the wild‐type plants, with obvious increase in plant height, stem diameter, internode length and number, leaf width and length, and shoot weight (Figures 5a–c, 6, S4a–c and Table S3). For further analyses of transgenic plants, propagated plantlets were grown in Urumqi (Xinjiang Province, China) for field trial in 2013 (transgenic trial permit number: 2012‐T03). Again, transgenic plants produced greater biomass, resulting in improved plant height and stem diameter at both breast height (DBH) and ground level (Figure 7a–e).

### Expression of *PtCYP85A3* increases the content of bioactive BR in transgenic plants

To understand whether PtCYP85A3 indeed functions in BR biosynthesis, we examined the endogenous levels of bioactive BRs in the juvenile leaves of 3‐month‐old WT and *PtCYP85A3* transgenic plants grown in glasshouse. Compared to the WT plants, the levels of 28‐norbrassinolide (28‐norBL), 28‐norcastasterone (28‐norCS), 28‐homobrassinolide (28‐homoBL), TY (typhasterol) and TE (teasterone), the common intermediates in BR‐biosynthetic pathway, were extremely low and undetectable. And no significant change was observed in the content of BL. However, the level of CS, the most abundant bioactive BR, was significantly higher in all transgenic lines (Table 1). These results suggest that overexpression of *PtCYP85A3* successfully enhanced the contents of bioactive BR in transgenic plants.

**Table 1:** BR content analyses

ng/g (FW)	CS	BL	28‐norBL	28‐norCS	28‐homoBL	TY	TE
WT	3.14 ± 0.10	0.34 ± 0.03	nd	nd	nd	nd	nd
L3	3.43 ± 0.06 **	0.32 ± 0.02	nd	nd	nd	nd	nd
L5	3.66 ± 0.09 **	0.32 ± 0.03	nd	nd	nd	nd	nd
L8	3.46 ± 0.12 **	0.38 ± 0.06	nd	nd	nd	nd	nd

Young leaves of 3‐month‐old wild‐type and transgenic poplar plants (lines 3, 5 and 8) grown in glasshouse were used. CS, castasterone; BL, brassinolide; 28‐norBL, 28‐norbrassinolide; 28‐norCS, 28‐norcastasterone; 28‐homoBL, 28‐homobrassinolide; TY, typhasterol; TE, teasterone. **indicates significant differences in comparison with WT at P < 0.01 (Student‘s t‐test).

### *PtCYP85A3* promotes xylem differentiation in transgenic plants

**Figure 8:**
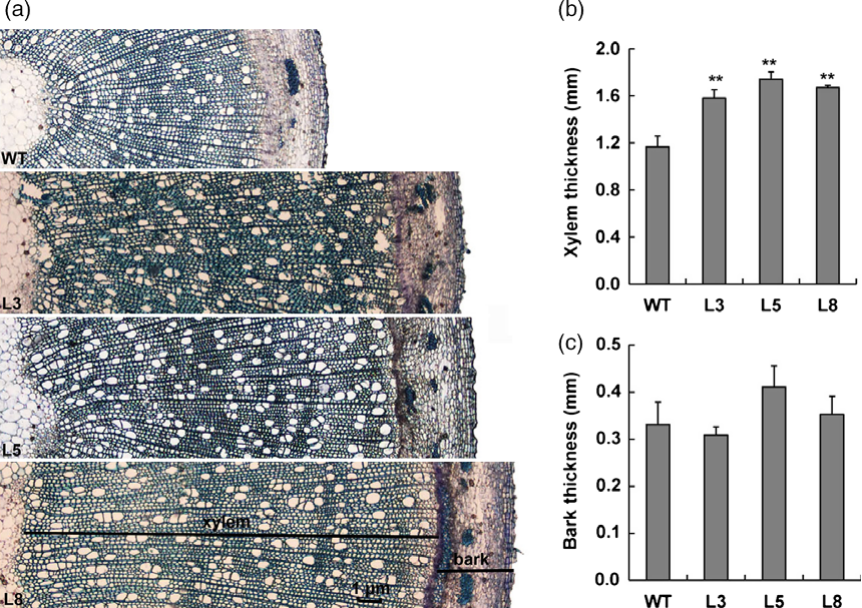
Effects of *PtCYP85A3* on xylem differentiation in transgenic poplar. (a) Cross sections of stems showing increased xylem area in 3‐month‐old wild‐type and transgenic plants (lines 3, 5 and 8). (b) Measurement of xylem and bark. ** indicates significant differences in comparison with WT at *P *< 0.01 (Student's *t*‐test).

**Figure 9:**
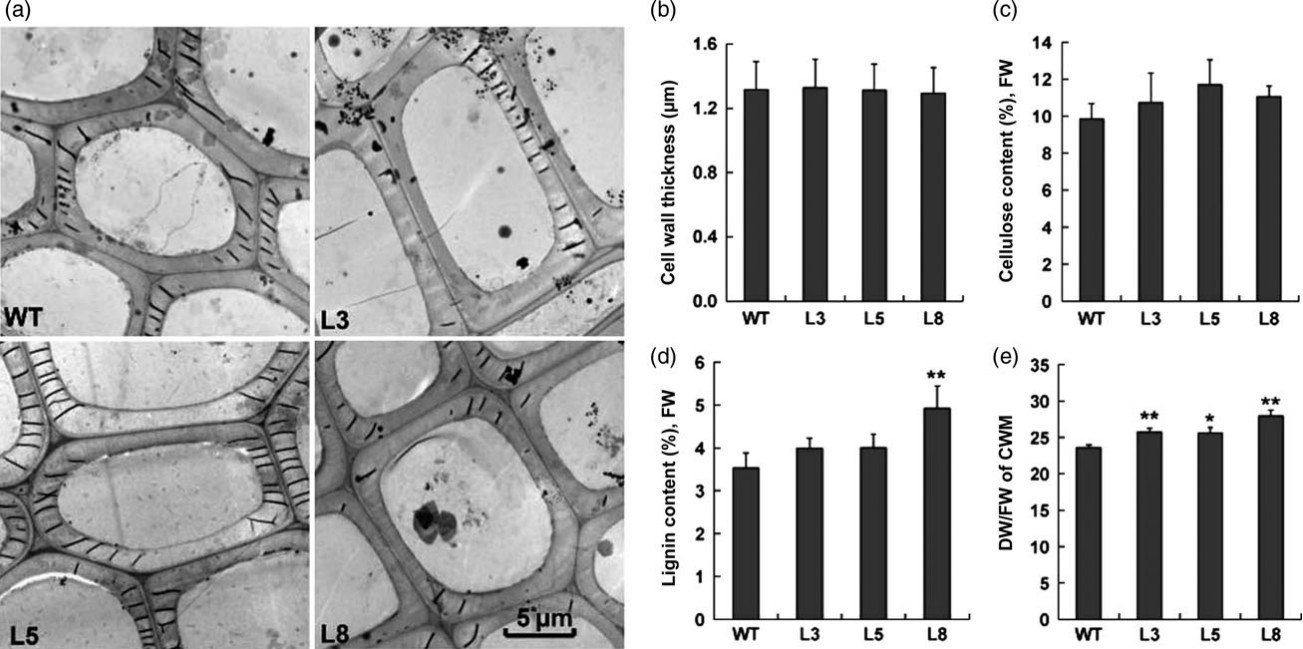
Secondary cell wall thickness analyses in the xylem of transgenic poplar. (a) Transmission electron micrographs of xylary fibres in the stems of wild‐type (WT) and transgenic plants (lines 3, 5 and 8). (b) Average values of cell wall thickness of xylem fibres. Values shown are means and SDs of 100 fibre cells from three plants. Scale bar = 5 μm. (c, d) Cellulose and lignin contents in the xylem tissues. Three individual plants were sampled for each transgenic line. FW, fresh weight. (e) Cell wall dry material weights in the stem of WT and transgenic plants. Error bars show SD (*n *= 3). * and ** indicate significant differences in comparison with WT at *P *< 0.05 and *P *< 0.01, respectively (Student's *t*‐test).

To further understand the potential function of PtCYP85A3 during the secondary growth of wooden plants, microscopic analyses were conducted with the stems of WT and transgenic plants. As shown in Figure 8, the growth of xylem was improved in all tested *PtCYP85A3* transgenic lines (Figure 8a). Transgenic plants produced more xylem (Figure 8b), although no significant difference was seen in the production of bark between WT and transgenic plants (Figure 8c). Transmission electron microscopy (SEM) analyses demonstrated that the thickness of secondary cell wall (Figure 9a, b) and the contents of cellulose and lignin (Figure 9c, d) were not remarkably affected in most of the transgenic lines (exempt the content of lignin in line L8 which was higher than the WT). But the dry weight of cell wall materials was obviously higher in all transgenic lines due to the increased xylem cells (Figure 9e). In addition, the percentage of long fibres (>500 μm) increased, whereas the percentage of short fibres (<400 μm) decreased in transgenic plants (Figure S5). These results suggest that *PtCYP85A3* can augment the number of xylem cells but does not affect xylem cell wall thickness and components during secondary cell wall growth in poplar.

### Up‐regulated expression of wood‐associated genes in transgenic plants

Previous studies have proved that the secondary growth of xylem cell wall is regulated by transcriptional factors for cell wall biosynthesis and modification (Zhong *et al*., 2008, 2011). To address the molecular basis for the increased growth and xylem production, we investigated the expression of genes encoding these transcriptional factors in the stems of both WT and transgenic plants. We found that three secondary cell wall‐associated transcription factor genes (*PtMYB2*,* PtMYB*18 and *PtMYB*20) and two secondary cell wall cellulose synthase genes (*PtCesA5* and *PtCesA17*) were significantly up‐regulated in the stems of transgenic lines (Figure S6).

## Discussion

Previous reports that AtCYP85A2 catalyses the biosynthesis of bioactive CS and BL and overexpression of *AtCYP85A2* improved the vegetative and reproductive growth in transgenic *Arabidopsis* suggest that CYP85A might be an important rate‐limiting enzyme in the BR biosynthesis pathway generally existed in various plant species (Kim *et al*., 2005). Indeed, CYP85A homologues have been identified in many plant species including *Oryza sativa* (Hong *et al*., 2002; Mori *et al*., 2002), *Vitis vinifera* (Symons *et al*., 2006), *Solanum lycopersicum* (Bishop *et al*., 1996, 1999; Nomura *et al*., 2005), *Pisum sativum* (Jager *et al*., 2007) and *Populus trichocarpa* (Kim *et al*., 2008; Pearce *et al*., 2002). To affirm whether *Populus CYP85A* gene family members have similar functions to *AtCYP85A2*, we identified three homologues in the *Populus* genome. As PtCYP85A3 shares the maximum sequence identity with AtCYP85A2, we cloned its encoding sequence and verified its functions in transgenic poplar. We demonstrate here that PtCYP85A3, a member of the P450 (CYP85A) gene family, is involved in BR biosynthesis and wood formation in poplar.

PtCYP85A3 shares very high amino acid sequence identity with the *Arabidopsis* AtCYP85A2 and tomato SlCYP85A1 and contains all the highly conserved domains (Figure 1a, b; Bishop *et al*., 1996, 1999; Kim *et al*., 2005; Nomura *et al*., 2005), suggesting its possible function as a putative cytochrome P450 monooxygenase in *Populus*. As *Populus* has been taken as an ideal model tree plant (Jansson and Douglas, 2007; Tuskan *et al*., 2006), a comparative study on the genetic functions of CYP85A gene family between *Arabidopsis* and *Populus* will provide direct evidence in understanding the mechanism of BR‐regulated plant growth and development between herbaceous and woody plants. We found that *PtCYP85A3* was predominantly expressed in young leaves (Figure 2a, d) and its expression was strongly suppressed by BL in poplar (Figure 2b, c, e, f). Therefore, the high expression in young leaves and the BR feedback‐regulated expression of *PtCYP85A3* could imply a pivotal role of PtCYP85A3 in BR metabolism and the normal growth of trees.

Based on the high sequence identity with AtCYP85A2 and SlCYP85A1, the biological function of PtCYP85A3 was first examined in *cyp85a2‐2* and *d* ^*x*^ mutants (Figure S1a‐c). The most distinctive phenotypic change in both mutants was the dwarfed shoot growth. *PtCYP85A3* successfully rescued the growth defect and restored their growth phenotype to that of the wild type, indicating that *PtCYP85A3* can be a functional allele of *AtCYP85A2*/*SlCYP85A1* in poplar. A mutant and curled leaf growth phenotype was also observed in the transgenic *cyp85a2‐2* and *d* ^*x*^ plants complemented with *PtCYP85A3* (Figure S1b‐c). This could be due to the excessive expression of *PtCYP85A3* driven by the strong CaMV 35S promoter. Similar phenotype was also reported with transgenic *bri1* complementary plants (Wang *et al*., 2001).

Plants with reduced BR‐biosynthetic enzymes or increased BR metabolism enzymes generally exhibit phenotypes such as severe dwarfism, round and dark‐green leaves, delayed senescence, reduced male fertility and defective skotomorphogenesis in darkness, whereas those with increased BR‐biosynthetic enzymes show larger leaves, increased growth and tolerance to biotic and abiotic stress. Engineering the synthetic and metabolic enzyme activity can change the endogenous levels of BRs for the fine regulated growth and development of plants. Unlike other plant hormones, BRs are not transportable between different tissues via long‐distance transport mechanisms (Symons and Reid, 2004). In addition, plant growth, including both shoots and roots, is promoted by low concentration of BRs, but inhibited by high concentration of BRs (Haubrick and Assmann, 2006). Therefore, it is crucial to maintain adequate physiological BR function by means of the equilibrium between BR biosynthesis and metabolism in plants.

To dissect the exact biological functions of *PtCYP85A3*, we constitutively expressed it in tomato and poplar (Figures 4a–e, 5a–c, 6, 7a–e, S2a–d, S3a–d, S4a–c; Tables S2, S3) and examined the endogenous BR contents (Table 1). We observed that *PtCYP85A3* transgenic plants showed improved growth, same as did the transgenic plants expressing *AtCYP85A2* (Kim *et al*., 2005) and *AtDWF4* (Choe *et al*., 2001; Wu *et al*., 2008). In transgenic rice expressing *AtDWF4*, the levels of 6‐deoxocathasterone (6‐DeoxoCT) and other downstream BR pathway intermediates increased obviously (Wu *et al*., 2008). In our study, most of the BR pathway intermediates tested were undetectable, except for the bioactive CS, which increased significantly (Table 1; Wu *et al*., 2008). This may be due to the tremendous species differences between perennial woody *Populus* and annual herbaceous rice, and the CYP85A family members, including their expression patterns (Figure 2a, d) and their functions in the metabolism of BRs (Choe *et al*., 2001; Kim *et al*., 2005).

Physiological, genetic and molecular studies have revealed the roles of plant hormones in xylogenesis and vascular tissue differentiation. Expression of some cell wall synthetic genes was reduced in BR synthetic mutants, such as *XETs* and *MERI5* in *dwf1* (Kauschmann *et al*., 1996; Xu *et al*., 1995), *KOR* in *det2* (Sato *et al*., 2001). And brassinazole (Brz) can suppress the development of secondary xylem of cress plants (*Lepidium sativum*; Nagata *et al*., 2001). BRs increased tracheary element differentiation in zinnia (*Zinnia elegans*; Yamamoto *et al*., 2001) and can also regulate the expression of *EXPANSINS* (*EXP*), *VND6* and *VND7* (Kubo *et al*., 2005; Nemhauser *et al*., 2004). Recently, BES1 was shown to bind to and regulate the activity of most of the PCW and SCW *CesA* promoters, except that of *CesA7* (Xie *et al*., 2011). Hussein's research also showed that *dim1* exhibited a dwarf phenotype with an up to 38% and 23% reduction in total lignin and cellulose, respectively (Hossain *et al*., 2012). All these results indicate that BRs could play a crucial role in cell wall biosynthesis and remodelling.

In this study, we found that consistent with the increased endogenous levels of CS, transgenic poplar plans produced more xylem than did the wild‐type plants (Figures 8a–c, 9a–e). In addition, although no significant changes in the secondary cell wall thickness, as well as in the cellulose and lignin contents, were observed between WT and transgenic plants, the percentage of long fibres (>500 μm) increased in transgenic plants (Figure S5). Further studies revealed that expressions of several cell wall cellulose synthase and secondary cell wall‐associated transcription factor genes were significantly up‐regulated in transgenic plants (Figure S6). These results suggest that *PtCYP85A3* can function in both secondary cell division and fibre elongation during wood formation in poplar. Taken together, *PtCYP85A3* is a functional allele of *AtCYP85A2* and *SlCYP85A1*. Overexpression of *PtCYP85A3* increased CS production and consequently promoted the growth and wood formation of transgenic plants. The remarkably increased growth and biomass production in transgenic plant shown in this work imply a great potential of *PtCYP85A3* for the molecular breeding of fast‐growing trees by manipulating BR production in aspen and other wooden plants.

## Experimental procedures

### Plant materials and growth conditions

The T‐DNA insertion *cyp85a2‐2* mutants were obtained from the *Arabidopsis* Biological Resource Center and the Nottingham *Arabidopsis* Stock Centre. *Arabidopsis* and *Solanum lycopersicum* (Micro‐TOM) seeds were sterilized with 10% sodium hypochlorite for 5 min and washed three times with sterilized water and then plated on MS medium (Murashige and Skoog, 1962) with 2% (w/v) sucrose and 0.8% (w/v) agar. For *Arabidopsis* plants grown in glasshouse, seeds were stratified at 4 °C for 2 days and then transferred to 22 °C for another 7 days before transferred to soil and then grown under 12 h of light/12 h of dark cycles in the glasshouse at 22 °C (light) or 19 °C (dark).

The aspen hybrid clone Shanxin yang (*Populus davidiana* × *P. bolleana*) was used for plant transformation. Poplar plantlets were amplified by aseptically transferring shoot apices to fresh MS medium with 0.1 mg/L naphthylacetic acid (NAA). Plantlets were grown in glass bottles in the culture room with cool white fluorescent light (~200 μmol m^−2^ s^−1^) under 12‐h light/12‐h dark photoperiod at 21–25 °C/15–18 °C (day/night). One‐month‐old plantlets were transferred to soil and kept in glasshouse under 14‐h photoperiod comprising natural daylight supplemented with lamps (120–150 μEm^−2^ s^−1^) at about 21–25 °C/15–18 °C (day/night). All plants were well watered according to the evaporation demands during different growth stages and fertilized biweekly with half strength of Hoagland nutrient solutions.

### Transgenic vector construction and plant transformation

To isolate candidate genes encoding DWARF enzyme from *Populus trichocarpa*, a blast search against the tomato DWARF protein sequence (NP_001234263) was performed in poplar genome database (http://www.phytozome.net/poplar). Three P450 members belonging to CYP85 clan were identified, and sequence alignment was carried out using the CLUSTALX program. We further isolated the coding sequence (CDS) of *PtCYP85A3* that is closest to the tomato DWARF by PCR amplification using gene‐specific primers (5′‐ATGGCAGTTCTCTTGATGGTTCTTG‐3′) and (5′‐TTAGTGAGATGAGACCCTAATGTGTAGC‐3′). A fragment of ~1.4 kb was cloned into the *Sma* I site of pBluescript II KS (pKS, Stratagene, La Jolla, CA), and three independent clones were selected for further sequence confirmation.

The plant expression vector pCAMBIA2301‐*PtCYP85A3* was constructed as described previously (Tang *et al*., 2010). The 1395‐bp *PtCYP85A3* coding sequence was inserted into a modified pCAMBIA2301or pCAMBIA1301 vector via the *Xba* I and *Pst* I restriction sites, under the control of cauliflower mosaic virus (CaMV) 35S promoter. The resultant construct was introduced into *Agrobacterium tumefaciens* strain GV3101 for *Arabidopsis* transformation or EHA105 strain for tomato and poplar transformation. *Arabidopsis* plants (wild type and *cyp85a2‐2*) were transformed by the floral dipping method (Clough and Bent, 1998). Transgenic plants were screened on MS medium supplemented with 30 μg/mL kanamycin or hygromycin for 7–10 days, and the survivors were transferred into soil for propagation. Micro‐Tom and the Ailsa Craig *d* ^*x*^ (LA2838) mutant were used for *Agrobacterium*‐mediated transformation as described previously (Zhang and Blumwald, 2001). Briefly, surface‐sterilized tomato seeds were placed on MS agar medium. Cotyledons of 1‐week‐old seedlings were aseptically cut into small pieces and then co‐incubated for 20 min with *Agrobacterium* cultures. Excess *Agrobacterium* culture was removed by sterilized filter paper, and the explants were transferred to co‐cultivation media. After 2 days at 24 °C, explants were transferred to shoot induction media containing 50 mg/L kanamycin. Once the regenerated shoots reached ~2–4 cm in length, they were transferred to rooting media. Tomato plantlets with well‐developed roots were planted in pots.

The Shanxin yang was transformed as described previously (Wang *et al*., 2011). Independently regenerated transgenic lines were propagated and transplanted into soil.

### GUS (β‐glucuronidase) staining

To confirm the expression of reporter (GUS) gene co‐transformed with *PtCYP85A3*, histochemical staining was conducted as described previously (Tang *et al*., 2012). Leaves were cut from poplar plantlets and incubated at 37 °C for 12 h in a buffer solution containing 0.1 M sodium phosphate buffer (pH 7.0), 0.5 mm ferricyanide, 0.5 mm ferrocyanide, 10 mm EDTA, 0.1% Triton X‐100 and 1 mm 5‐bromo‐4‐chloro‐3‐indolyl‐β‐D‐glucuronide (X‐Gluc). The stained leaves were cleared of chlorophyll with 75% ethanol.

### Plant treatments, RT‐PCR and quantitative real‐time PCR analyses

For expression pattern analysis of *PtCYP85A3* in poplar, total RNA was extracted with the RNAiso reagent (Takara, Japan) from different organs or tissues of 3‐month‐old Shanxin yang, including apical buds (A), juvenile leaves (JL), mature leaves (ML), petiole (Pe), phloem of elongating stem (EP), xylem of elongating stem (EX), phloem of thickening stem (TP), xylem of thickening stem (TX) and roots (R). After treated with DNase I (Promega), an amount of 2 μg of total RNA was subjected to reverse transcription reaction using the reverse transcriptase ReverTra Ace (TOYOBO, Japan) at 42 °C for 1 h. The resultant cDNA was then used for RT‐PCR and quantitative real‐time RT‐PCR with gene‐specific primers (Table S1). The elongation factor gene *PtEF1*β was employed as an internal control (*PtEF1*β*‐RT‐F* and *PtEF1*β*‐RT‐R*; Table S1). Quantitative real‐time RT‐PCR analysis was performed with the Rotor‐Gene 3000 system (Corbett Research) using the SYBR Green Real‐time PCR Master Mix (TOYOBO, Japan) to monitor double‐stranded DNA products. Data analysis was performed with Rotor‐Gene software version 6.0, and relative amounts of mRNA were calculated based on the comparative threshold cycle method. The relative expression of each target gene was normalized using the housekeeping gene *PtEF1*β and the expression value of TP was set to 1.

For BL treatment, 3‐week‐old micropropagated plantlets were sprayed with 100 nm BL for indicated time periods or with different concentrations of BL for 30 min. The relative expression of *PtCYP85A3* was normalized using the housekeeping gene *PtEF1*β and the expression value of that at 0 min or treated with 0 nm BL was set to 1.

For expression analysis of *PtCYP85A3* in transgenic plants, the stem of 3‐month‐old transgenic plants grown in the glasshouse was used for RNA extraction. *PtCYP85A3*‐specific primers (*RT‐F* and *RT‐R*) were used for RT‐PCR analyses (Table S1). For expression analysis of the poplar wood‐associated genes in transgenic plants, the stem of 2‐month‐old wild‐type (WT) and transgenic plants (lines 3 5 and 8) grown in the glasshouse was used. Gene‐specific primers used were shown in Table S1.

### Subcellular localization study of PtCYP85A3

To determine the subcellular localization of PtCYP85A3 protein, *PtCYP85A3* gene sequence without the stop codon was in‐frame fused upstream to the YFP sequence in the pA7‐YFP vector. The resultant PtCYP85A3‐YFP, together with the control plasmid pA7‐YFP and the ER marker ER‐YFP (Nelson *et al*., 2007), was transfected into poplar mesophyll protoplasts, respectively. Co‐transformation of ER‐CFP in combination with PtCYP85A3‐YFP or YFP alone was also performed. Protoplast isolation and transformation were performed essentially according to Yoo *et al*. (2007). Fluorescence of YFP and CFP in the transformed protoplasts was imagined using a confocal laser scanning microscope (LSM510, Carl Zeiss) after the protoplasts were incubated at 23 °C for 16 h. For excitation of fluorescence proteins and chlorophyll, the following lines of argon ion laser were used: 514 nm for YFP, 458 nm for CFP and 488 nm for chlorophyll. Fluorescence was detected at 530–600 nm for YFP, 475–525 nm for CFP and 650 nm for chlorophyll.

### Anatomical observations

For histological observations, fresh stems from the same parts of 3‐month‐old wild‐type and transgenic plants grown in glasshouse were fixed with 2% formaldehyde and subsequently passed over a graded ethanol series. Then, the sections were embedded in paraffin. Eight‐micrometre‐thick sections were cut out with a rotary microtome. After the paraffin was removed, sections were stained with 0.05% toluidine blue and examined with a light microscope. Images were captured under bright field using an ECLIPSE 80i microscope (Nikon, Tokyo, Japan). The radial widths of phloem, cambium and xylem were measured using the Image Tool software (UTHSCSA, Texas).

For transmission electron microscopic observation, the middle stems of 2‐month‐old plants grown in the glasshouse were fixed in glutaraldehyde solution and embedded in EPON 812 resin (Shell, New York). Sections (80–70 nm) were cut, poststained with uranyl acetate and lead citrate, and observed with an H‐7650 electron microscope (HITACHI, Tokyo, Japan). The secondary cell wall thicknesses of the xylem fibres were measured using the UTHSCSA Image Tool software.

### BR content assays

Plant tissues (1‐g FW young leaves) from 3‐month‐old wild‐type and transgenic plants (lines 3, 5 and 8) grown in glasshouse were frozen in liquid nitrogen and grounded into fine powder with a mortar and pestle and then transferred into a 10‐mL centrifuge tube. Stable isotope‐labelled BRs [^2^H_3_]BL (2 ng) and [^2^H_3_]CS (2 ng) were added into the mixture followed by extraction with acetonitrile (5 mL/g) overnight at −20 °C. Then, the samples were preprocessed employing double‐layered solid phase extraction (DL/SPE) combined with boronate affinity polymer monolith microextraction (BA/PMME). Finally, BRs were determined by liquid chromatography–mass spectrometry as described previously (LC‐MS, Ding *et al*., 2014).

### Cellulose and lignin content determination

The stems of 3‐month‐old WT and transgenic plants (lines 3 5 and 8) grown in the glasshouse were cut into small pieces, ground to fine powder in liquid nitrogen and dried at 70 °C. Cell wall material (CWM) isolation was performed as described previously (Foster *et al*., 2010a) by sequentially washing the samples with 70% (v/v) ethanol, chloroform : methanol (1 : 1) and acetone. Starch was removed from the pellet by incubation in 1 mL of a 0.1 M sodium acetate buffer (pH 5.0) with amylase (50 μg/1 mL H_2_O; from *Bacillus* species, Sigma, St Louis) and pullulanase (from *Bacillus acidopullulyticus*; Sigma, St Louis) at 37 °C for 12 h. After washed three times with water, the resultant CWM was suspended with acetone and dried at 35 °C for 12 h. Cell wall materials were used to determine the contents of cellulose and lignin.

To determine the cellulose content, CWM was incubated in 1 mL of Updegraff reagent for 30 min at 100 °C and then washed three times with 1 mL of acetone. The pellet (crystalline cellulose) was completely hydrolysed into glucose in 175 μL of 72% sulphuric acid at room temperature for 45 min. After the addition of 825 μL water, 10 μL of each sample supernatant and 90 mL of water were pipetted into separate cells of 96‐well polystyrene microtitre plates before the addition of 200 μL of freshly prepared anthrone reagent. The plate was heated for 30 min at 80 °C, and the absorption at 625 nm was measured at room temperature (Foster *et al*., 2010a).

To determine the lignin content, CWM was incubated in 100 μL of freshly made acetyl bromide solution (25% v/v acetyl bromide in glacial acetic acid) for 2 h at 50 °C and then heated for an additional hour with vortexing every 15 min. After being cooled on ice to room temperature, 400 μL of 2 m sodium hydroxide and 70 μL of freshly prepared 0.5 m hydroxylamine hydrochloride were added and mixed. Then, 1.43 mL glacial acetic acid was added. The solution (200 μL) was pipetted into the UV‐specific 96‐well plates and was read in an ELISA reader at 280 nm (Foster *et al*., 2010b).

### Fibre length measurements

Fibre lengths were measured as described previously (Wang *et al*., 2013). Trimmed xylem pieces from the middle part of stems of 3‐month‐old WT and transgenic plants grown in glasshouse were prepared. The samples were bathed in a solution of 10% hydrogen peroxide and 50% glacial acetic acid for 4–6 h at 95 °C, rinsed with distilled water for three times, neutralized with sodium carbonate and washed again with distilled water. Finally, fibres were separated from each other in distilled water and measured under an ECLIPSE 80i microscope (Nikon, Tokyo, Japan). The lengths of 300 fibres from three plants of wild type and each transgenic line were measured.

### Statistical analysis

For statistical analyses, Student's *t*‐test was used to generate every *P* value. All the tests were two‐tailed. The data were normalized, and all samples were normally distributed with homogeneity of variance. Sequence data from this article can be found in the GenBank data library under the following accession numbers: *AtCYP85A2* (AB087801), *PtEF1*β (eugene3.00091463), *PtCYP85A3* (EEF10243), *PtC*esA5 (AY055724), *PtCesA17* (XM_002325086), *PtMYB2* (XM_002299875), *PtMYB18* (XM_002305179), *PtMYB20* (XM_002313267).

## Conflict of interest

The authors declare no conflict of interest.

## Supporting information


**Figure S1** PtCYP85A3 can completely complement the *Arabidopsis* (*cyp85a2‐2*) and tomato (*d* ^*x*^) mutants.
**Figure S2** Molecular analyses of *PtCYP85A3* transgenic tomato.
**Figure S3** Phenotypes of wild type and *PtCYP85A3* transgenic plants (lines 3, 5 and 8).
**Figure S4** Growth comparison of wild type and *PtCYP85A3* transgenic plants grown in greenhouse.
**Figure S5** Percentages of xylem fibre lengths in WT, transgenic lines 3, 5 and 8.
**Figure S6** Expression analysis of secondary cell wall synthesis‐related MYB transcription factor and cellulose synthase genes.
**Table S1** Primers used in this study.
**Table S2** Overexpression of *PtCYP85A3* in the miniature tomato Micro‐Tom promotes shoot elongation, plant size and overall yield.
**Table S3** Overexpression of *PtCYP85A3* in poplar promotes biomass production.
