# Supplementary material for: Overexpression of Populus trichocarpa CYP85A3 promotes growth and biomass production in transgenic trees
Source: Plant Biotechnol J. 2017 Jun 17;15(10):1309–21. doi: 10.1111/pbi.12717 (PMC5595715; doi:10.1111/pbi.12717)
Supplement: Supplementary file 1 — Figure S1 PtCYP85A3 can completely complement the Arabidopsis (cyp85a2‐2) and tomato (d x ) mutants. Figure S2 Molecular analyses of PtCYP85A3 transgenic tomato. Figure S3 Phenotypes of wild type and PtCYP85A3 transgenic plants (lines 3, 5 and 8). Figure S4 Growth comparison of wild type and PtCYP85A3 transgenic plants grown in greenhouse. Figure S5 Percentages of xylem fibre lengths in WT, transgenic lines 3, 5 and 8. Figure S6 Expression analysis of secondary cell wall synthesis‐related MYB transcription factor and cellulose synthase genes. Table S1 Primers used in this study. Table S2 Overexpression of PtCYP85A3 in the miniature tomato Micro‐Tom promotes shoot elongation, plant size and overall yield. Table S3 Overexpression of PtCYP85A3 in poplar promotes biomass production. [file PBI-15-1309-s001.doc]

**Supporting Information**


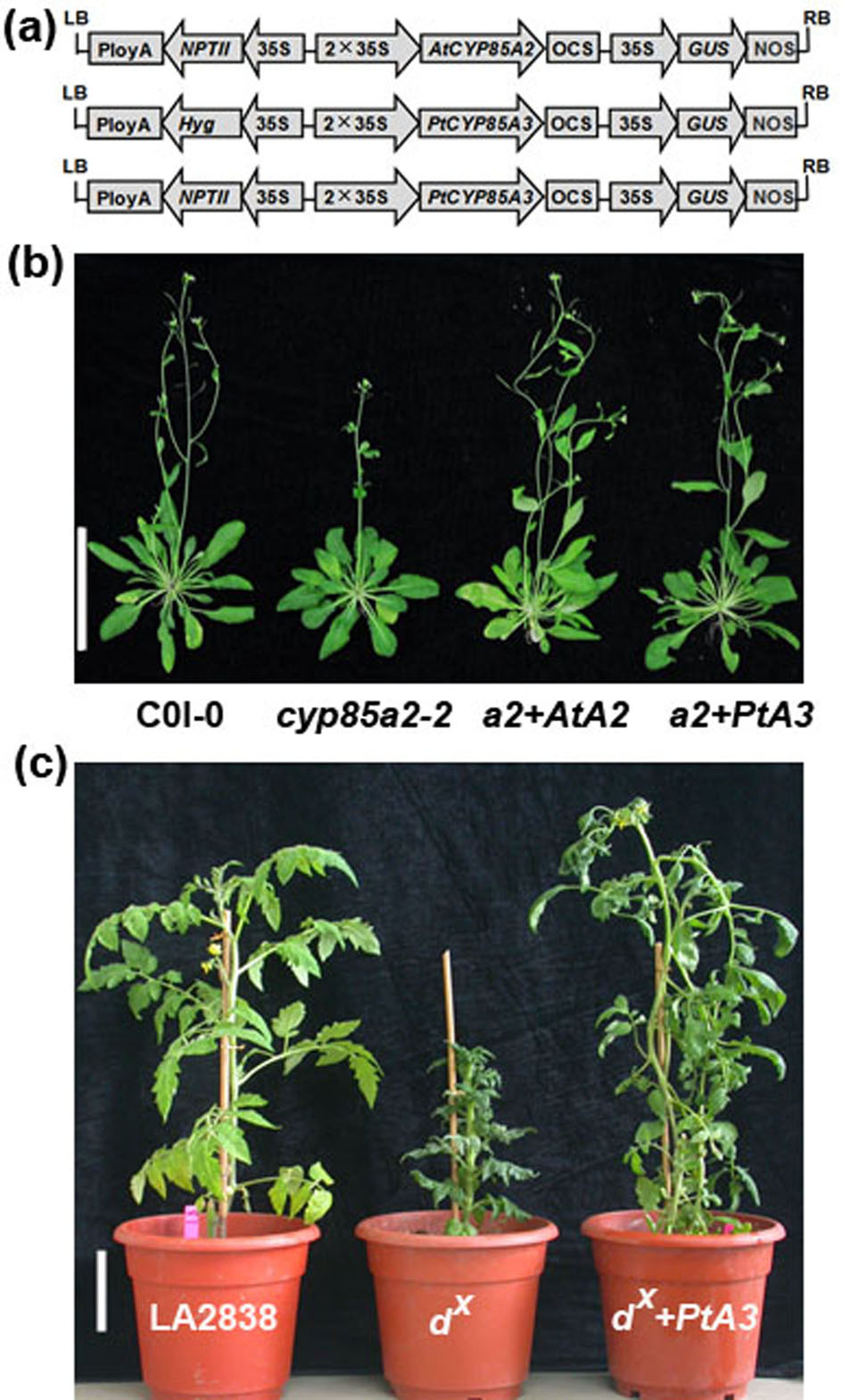


**Figure S1.** PtCYP85A3 can completely complement the *Arabidopsis* (*cyp85a2-2*) and tomato (*d^x^*) mutants. (a) Schematic map of pCAMBIA1301/2301-*PtCYP85A3* and pCAMBIA2301-*AtCYP85A2* constructs. Expression of *PtCYP85A3* and *AtCYP85A2* is driven by the cauliﬂower mosaic virus 35S promoter. (b) Phenotype of wild type *Arabidopsis* (Col-0), *cyp85a2-2* mutant, and transgenic *cyp85a2-2* plants complemented with *AtCYP85A2* (*a2*+*AtA2*) and *PtCYP85A3* (*a2*+*PtA3*)*.* Bar=10 cm. (c) Phenotype of wild type tomato (LA2838), *d^x^* mutant and *d^x^* transgenic plant complemented with *PtCYP85A3*. Bar=10 cm.


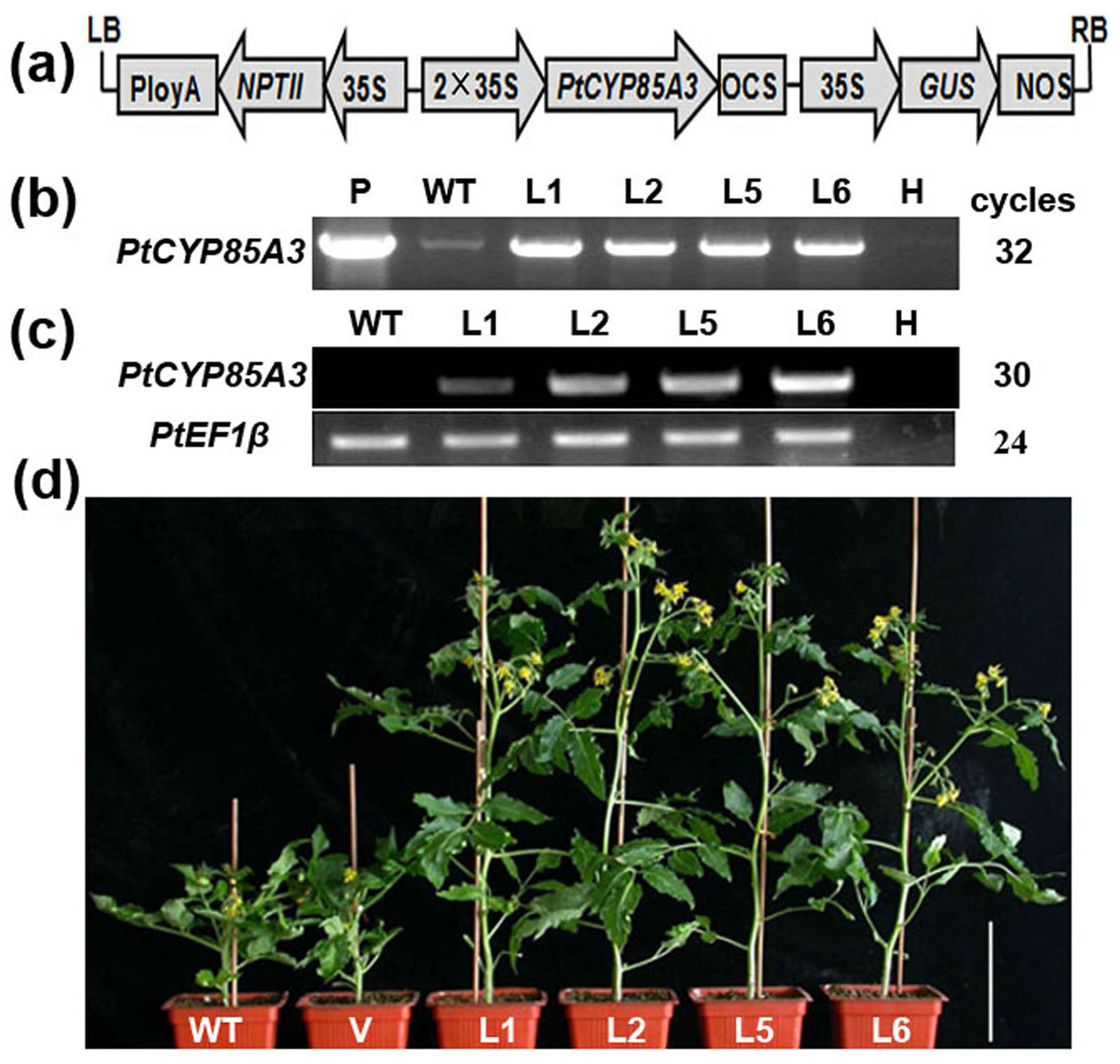


**Figure S2.** Molecular analyses of *PtCYP85A3* transgenic tomato. (a) Schematic map of pCAMBIA2301-*PtCYP85A3* construct. Expression of *PtCYP85A3* is driven by the cauliﬂower mosaic virus 35S promoter. (b) PCR analysis of wild type and four independent transgenic lines. P: plasmid; H: water; WT: wild type; L1-6: different transgenic lines. (c) RT-PCR analysis of wild type and four independent transgenic lines. H: water; WT: wild type; L1-6: different transgenic lines. (d) Phenotypes of wild type, vector control and different transgenic T_2_ seedlings (8 weeks). All transgenic plants showed elongated shoots and bigger plant size. Bar=10 cm.


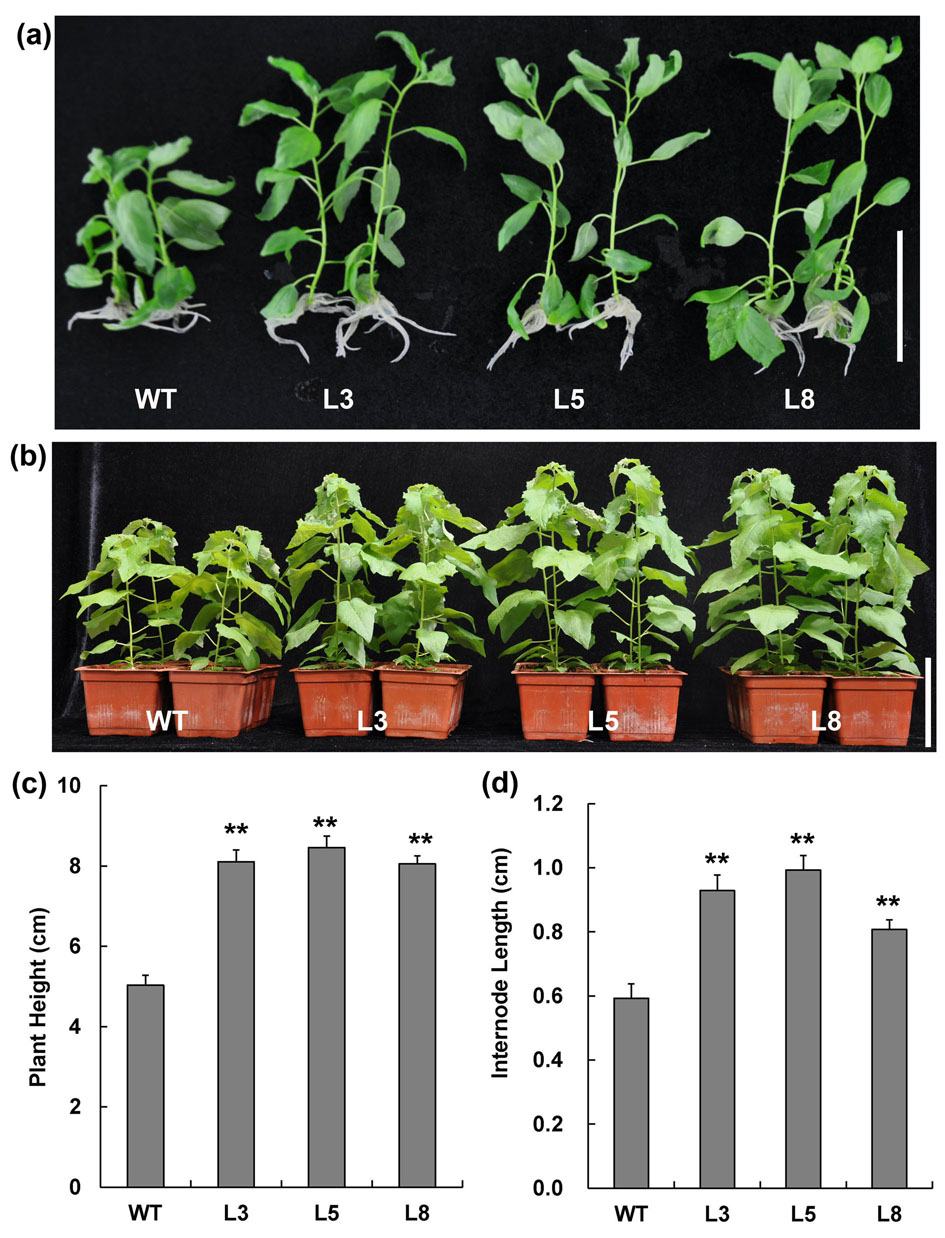


**Figure S3.** Phenotypes of wild type and *PtCYP85A3* transgenic plants (lines 3, 5 and 8). (a) One-month-old seedlings cultured on MS medium. Bar=5 cm. (b) One-month-old plants grown in greenhouse. Bar=10 cm. (c, d) Statistical analysis of plant height and internode length in (a). Error bars show SD (n≥15). Data are shown as means ± s.e.m. ** indicates significant difference between WT and transgenic plants under the same conditions at **P < 0.01 using the Student’s *t*-test (n = 3).


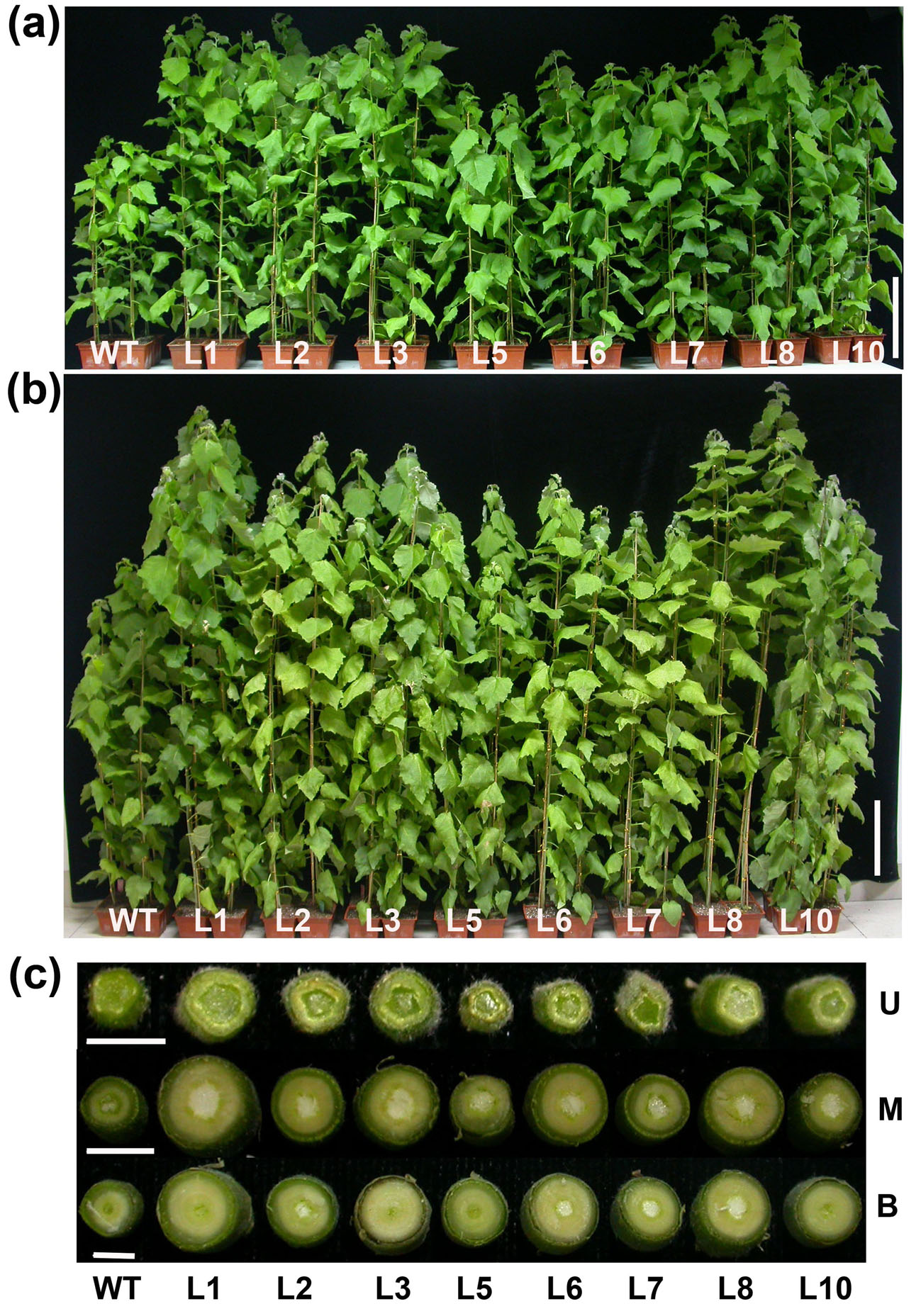


**Figure S4.** Growth comparison of wild type and *PtCYP85A3* transgenic plants grown in greenhouse. (a) Plant grown for nine weeks. Bar=20 cm. (b) Plant grown for 16 weeks. Bar=20 cm. (c) Stem cross sections to show the diameters of wild type and transgenic plants in (b). U, stem samples from the upper position; M, stem samples from the middle position; B, stem samples from the basal position. Bar =5 mm.


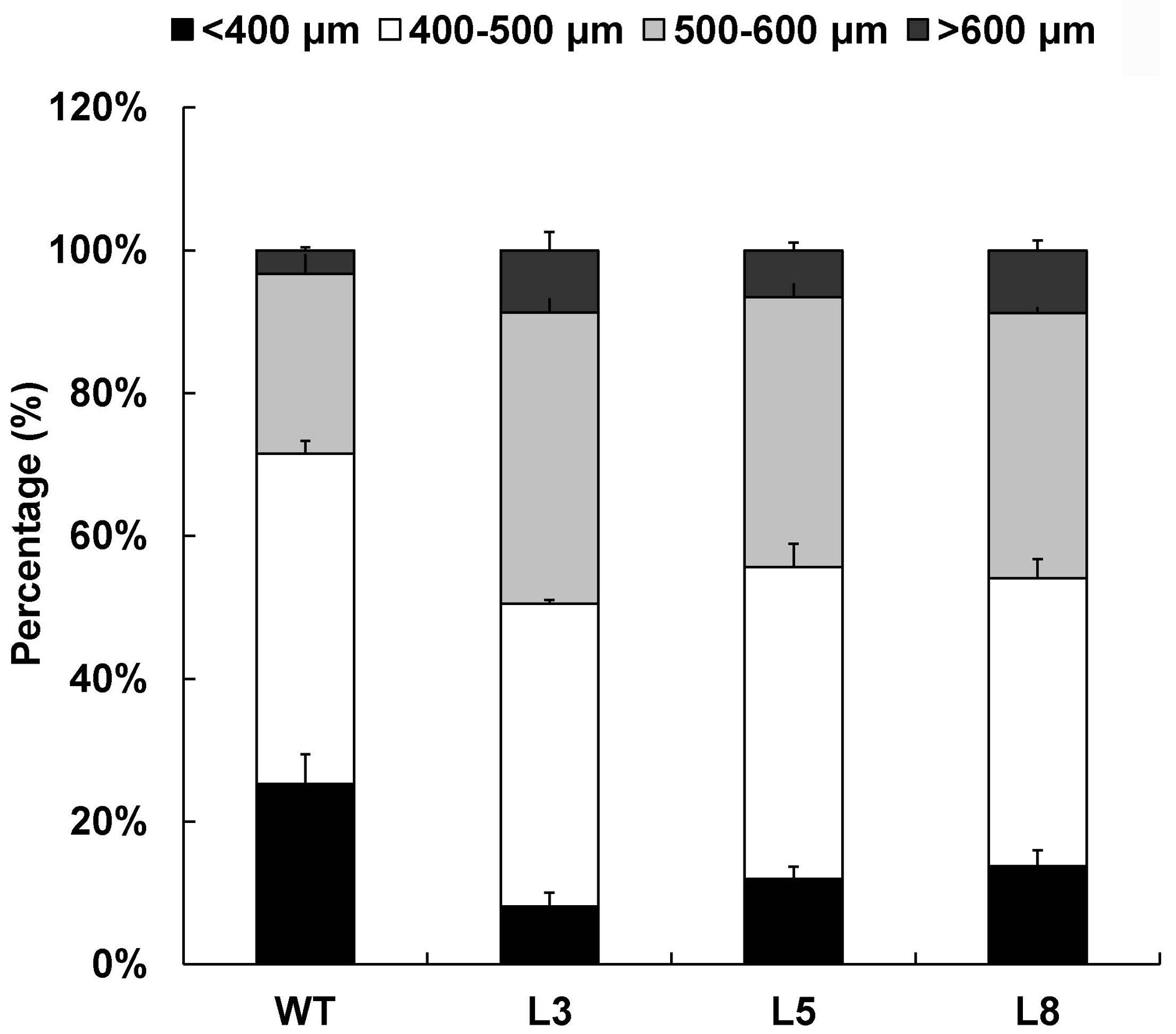


**Figure S5.** Percentages of xylem fiber lengths in WT and transgenic lines 3, 5 and 8. Values shown are means and SDs of 300 fiber cells from three plants of WT and each transgenic line, respectively.


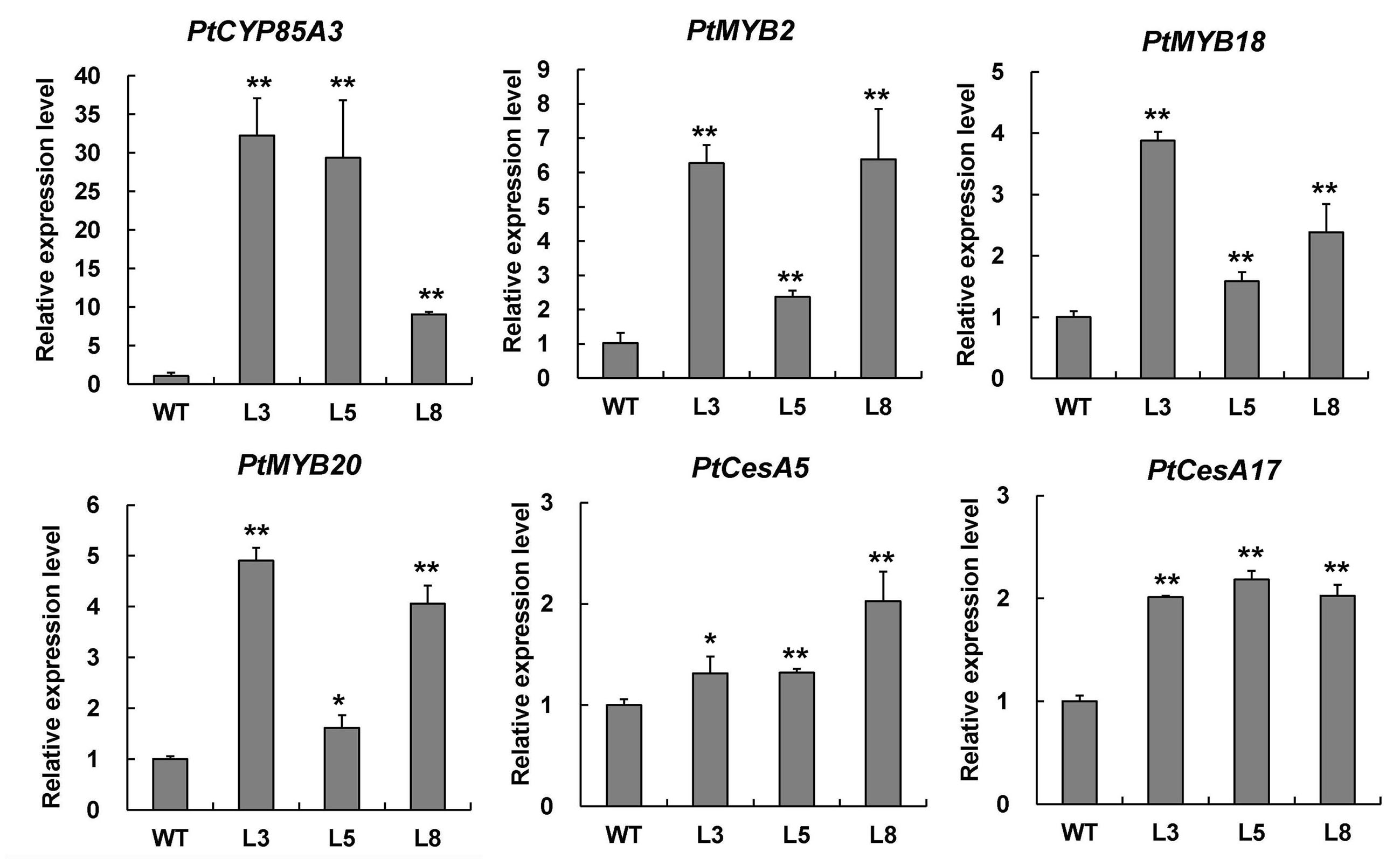


**Figure S6.** Expression analysis of secondary cell wall synthesis-related MYB transcription factor and cellulose synthase genes. Total RNA was isolated from the stems of three-month-old greenhouse grown plants. The elongation factor gene *PtEF1β* was employed as an internal control. The expression level of these genes in the stem of WT was set to 1. Error bars represent the SDs from three biological replicates. * and ** indicate significant differences in comparison to WT at *P* < 0.05 and *P* < 0.01, respectively (Student’s t-test).

**Table S1.** Primers used in this study.

| **Primer name** | **DNA sequence** |
| --- | --- |
| *PtEF1β-RT-F* | 5'-GACAAGAAGGCAGCGGAGGAGAG-3' |
| *PtEF1β-RT-R* | 5'-CAATGAGGGAATCCACTGACACAAG-3' |
| *PtCYP85A3-CDS-F* | 5'-ATGGCAGTTCTCTTGATGGTTCTTG-3' |
| *PtCYP85A3-CDS-R* | 5'-TTAGTGAGATGAGACCCTAATGTGTAGC-3' |
| *PtCYP85A3-RT-F* | 5'-CGCAATTAGAGAAAAGAAAAGGCCTGAG-3' |
| *PtCYP85A3-RT-R* | 5'-CCTTTCCTGGACACTGCCTGGTACCT-3' |
| *2*35S* | 5'-TTCGCAAGACCCTTCCTC-3' |
| *AtActin2-RT-F* | 5'-GGAAGGATCTGTACGGTAAC-3' |
| *AtActin2-RT-R* | 5'-GGACCTGCCTCATCATACT-3' |
| *AtCYP85A2-CDS-F* | 5'-CGGGATCCATGGGCATAATGATGATGATTTTG-3' |
| *AtCYP85A2-CDS-R* | 5'-GCGTCGACTCAGTAAGGTGAACACTTAAGATGG -3' |
| *AtCYP85A2-RT-F* | 5'-GAGTCCAAGCAAGAAACAACATCGATAG-3' |
| *AtCYP85A2-RT-R* | 5'-CTCCCTTATAGCCAAATGTTCTCTTCTGA-3' |
| *PtCesA5-RT-F* | 5'-ctgcaaacacagtcttgtccatcc-3' |
| *PtCesA5-RT-R* | 5'-agcttcgaaagtaagcatggctgc-3' |
| *PtCesA17-RT-F* | 5'-CTCTAGTCACGGGCAACACACTTT-3' |
| *PtCesA17-RT-R* | 5'-GTGCACATTGAAGCACCATCGTCA-3' |
| *PtMYB2-RT-F* | 5'-TTGGAGTGATGTAGCAAGGAA-3' |
| *PtMYB2-RT-R* | 5'-GATGAAGATGACAGTGACGGAT-3' |
| *PtMYB18-RT-F* | 5'-CAATGTTGCTGGAGAGCTGTT-3' |
| *PtMYB18-RT-R* | 5'-CAGGTTGATGAAGGAGAAGGTC-3' |
| *PtMYB20-RT-F* | 5'-ACACTTCCACATCCTCACCAAAT-3' |
| *PtMYB20-RT-R* | 5'-TGCACCAGTCACATCATAGCG-3' |

**Table S2.** Overexpression of *PtCYP85A3* in the miniature tomato Micro-Tom promotes shoot elongation, plant size and overall yield.

| Line | WT | Vector | L1 | L2 | L5 | L6 |
| --- | --- | --- | --- | --- | --- | --- |
| Plant Height (cm) | 14.80±2.45 | 14.53±1.66 | 32.10±4.48** | 35.70±3.23** | 33.68±3.77** | 32.52±4.97** |
| 1st Internode (cm) | 1.28±0.46 | 1.70±0.72 | 3.95±0.67** | 4.13±0.74** | 3.88±0.48** | 2.62±0.91** |
| 2nd Internode (cm) | 2.02±0.70 | 2.50±0.72 | 5.13±1.04** | 5.78±1.44** | 5.72±1.08** | 4.65±0.95** |
| Petiole Length (cm) | 2.50±0.38 | 2.81±0.30* | 4.29±0.87** | 5.93±1.43** | 5.18±0.67** | 4.73±0.93** |
| Shoot Fresh Weight (g) | 23.42±4.75 | 23.20±10.33 | 30.44±9.73 | 31.05±8.80 | 44.57±9.63** | 43.88±8.11** |
| Number of Flowers | 19.67±2.73 | 19.00±5.29 | 30.67±6.06** | 30.17±8.30* | 32.83±4.92** | 27.50±6.35* |
| Number of Fruits | 16.00±3.57 | 19.00±5.89 | 22.67±6.74 | 24.50±2.35** | 26.50±3.08** | 23.33±8.26 |
| Yield (g) | 37.25±11.57 | 36.79±14.71 | 52.11±22.88 | 60.30±6.39** | 56.90±16.13* | 44.09±25.91 |
| Weight per Fruit (g) | 2.29±0.28 | 1.94±0.48 | 2.32±0.69 | 2.49±0.39 | 2.13±0.38 | 1.82±0.49 |

Wild type and transgenic tomato plants were grown in greenhouse for two month. The height of plants, the length of the first and second internodes from the apex, and the length of petiole of wild type and transgenic tomato plants were quantified. Shoot fresh weights, as well as fruit numbers and yields were also determined at the harvest stage after three months. Numbers of the flowers were counted in the early reproductive stage. Values are mean± SD (n =12).

**Table S3. Overexpression of *PtCYP85A3* in poplar promotes biomass production.**

| **Line** | **WT** | **L1** | **L2** | **L3** | **L5** | **L6** | **L7** | **L8** | **L10** |
| --- | --- | --- | --- | --- | --- | --- | --- | --- | --- |
| **Height**  **(cm)** | 85.30+5.98 | 142.50+4.38  ** | 127.40+3.88  ** | 125.70+5.09  ** | 121.00+7.42  ** | 117.90+3.48  ** | 114.50+1.77  ** | 135.90+10.01** | 121.50+3.88  ** |
| **Stem Diameter**  **(mm)** | 3.74+0.36 | 6.27+0.17** | 4.88+0.25* | 5.32+0.25** | 4.41+0.17 | 5.24+0.34** | 4.98+0.27** | 5.28+0.20** | 4.64+0.15** |
| **Number of internode** | 36.40+2.66 | 48.20+2.70** | 46.60+1.60** | 46.00+1.84* | 42.00+0.71 | 43.00+1.70* | 43.20+2.04 | 48.40+2.36** | 46.00+1.17** |
| **Length of internode**  **(cm)** | 2.01+0.07 | 2.48+0.05 ** | 2.27+0.07 * | 2.22+0.05 * | 2.30+0.06 ** | 2.30+0.11 * | 2.18+0.09 | 2.25+0.11 | 2.28+0.07 * |
| **Stem biomass**  **(g)** | 8.68+2.23 | 30.48+1.70** | 16.13+1.83* | 19.74+0.65** | 13.19+0.98 | 18.95+1.71** | 13.24+1.36 | 21.87+2.97** | 16.72+1.26** |
| **Leaf length**  **(cm)** | 11.05+0.21 | 12.28+0.37* | 12.67+0.29** | 11.84+0.28* | 12.25+0.44* | 11.65+0.26 | 12.00+0.49 | 12.07+0.34* | 12.93+0.15** |
| **Leaf width**  **(cm)** | 10.74+0.14 | 11.89+0.27** | 11.81+0.33** | 11.10+0.23 | 11.87+0.37* | 11.28+0.17* | 11.29+0.34 | 11.55+0.34* | 11.96+0.19** |
| **Petiole**  **(cm)** | 3.84+0.05 | 4.44+0.10** | 4.79+0.25** | 4.36+0.08** | 4.79+0.14** | 4.14+0.10* | 4.10+0.16 | 4.18+0.19 | 4.88+0.11** |

Wild type and eight independent *PtCYP85A3* transgenic lines were grown in greenhouse. Plant height, stem diameter, shoot biomass, internode number and length, leaf length and width, and petiole length from sixteen-week-old plants were quantified. Values are mean± SD (n≥5). * and ** indicate significant differences in comparison to WT at *P* < 0.05 and *P* < 0.01, respectively (Student’s t-test).
